# Autism's heterogeneity in historical perspective: from challenge to opportunity

**DOI:** 10.3389/fpsyg.2023.1188053

**Published:** 2023-08-03

**Authors:** Marga Vicedo

**Affiliations:** Institute for the History and Philosophy of Science and Technology, University of Toronto, Toronto, ON, Canada

**Keywords:** autism, AS, heterogeneity of autism, history of autism, Lancet Commission on autism, Asperger, Kanner

## Introduction

Recently, the Lancet Commission on the Future of Care and Clinical Research in Autism introduced the concept of “profound autism” in a report signed by 32 representatives from ten countries. Concerned about addressing autistic people' diverse needs, the Commission suggests using “profound autism” as an “administrative term” to designate autistic adult individuals who cannot take care of basic daily needs on their own (Lord et al., 2022). But in a February 14, 2022 open letter, over 20 organizations of autistic people from different countries criticized this label as “highly problematic” (Open letter to the Lancet Commission on the future of care clinical research in autism, 2022). I believe that both positions can be fully understood in light of the history of autism.

To understand the Commission's goals as well as the current fears about the division of autism in different groupings, we must consider the complex history of the psychological categorization and moral valuation of autistic people. In my view, this history also shows that addressing the heterogeneity of autism is a crucial scientific need and ethical imperative today.

## Autism: from symptom to spectrum

The concept of autism has evolved over time and the heterogeneity of the condition has been at the center of that evolution. The diversity among the individuals diagnosed as autistic over the years has been a key concern in scientific and social discussions since the postulation of two forms of autism in the 1940s.

In 1911, the Swiss psychiatrist Eugen Bleuler introduced the term “autistic” to refer to the tendency of some patients to self-isolate and he presented this tendency as a characteristic of schizophrenia (Bleuler, 1911). Following Bleuler, child psychiatrists began to consider autistic behavior as a symptom of childhood schizophrenia. However, Russian psychiatrist Grunya Sukhareva (also Ssucharewa) pointed out that many children who displayed autistic behavior did not become schizophrenic; on the contrary, they improved over time. She also noted that some of them had impressive musical or numerical skills (Ssucharewa, 1926, 1932). Thus, these children did not fit under either of the two major categories used to classify those whose behavior deviated from standard notions of normality at the time: mental illness (such as schizophrenia), or cognitive disability (commonly named “feeblemindedness”). The need for new categories also became apparent to pediatrician Hans Asperger and child psychiatrist Leo Kanner in the late 1930s.

Asperger postulated the existence of what he called “autistic psychopathy.” Psychopathy then referred in the psychiatric literature to a “personality type.” Individuals categorized as psychopaths were troublesome in social settings due to interpersonal difficulties. Asperger was building upon the work of Anni Weiss and Georg Frankl, his former colleagues at the University of Vienna's therapeutic pedagogy ward. Weiss and Frankl had noticed some children who could not establish “emotional contact” with others because of their inability to grasp the affective tone of language. For them, these children were born with a disturbance of the social instincts (Frankl, 1934; Weiss, 1935). In his first and little known 1938 paper on the topic, Asperger separated autistic psychopaths in two groups. One included autistic children with limitations in the social realm who nevertheless displayed high intelligence and remarkable skills in specific areas. Asperger said that many of them would become scientists who could make important contributions to society. Children in the other group displayed useless and eccentric interests, had cognitive difficulties, and sometimes ended up developing schizophrenia later in their lives (Asperger, 1938). However, in his better known 1944 paper on “Autistic Psychopathy in Childhood,” Asperger did not mention the group of children with cognitive disabilities (Asperger, 1944).

Around the same time, working at John Hopkins University Medical School, Kanner presented “early infantile autism” as a new syndrome. Starting in 1938, Kanner puzzled over some children brought to his clinic who shared behavioral similarities but did not fit into accepted psychiatric categories. After Weiss and Frankl escaped Nazi persecution and ended up in Baltimore, Kanner also benefited from their insights about the children they had seen in Vienna (Vicedo and Ilerbaig, 2020). In 1943 Kanner described 11 children displaying “autistic aloneness,” “insistence on sameness,” and limited emotional engagement with people. For Kanner, they suffered from an affective disorder. He noted that though most of these children had difficulties with language, they possessed good cognitive “potentialities” (Kanner, 1943). A year later, he named their condition “infantile autism” (Kanner, 1944). Kanner first thought autism was inborn since it was manifested in infancy; however, his focus on autism's affective aspects later led him to seek its cause in a cold home environment and especially what others labeled “refrigerator mothers” (Vicedo, 2021).

Asperger and Kanner worked at a time when diagnosing a child with a psychopathological condition often had catastrophic consequences. After the annexation of Austria into the German Reich in 1938 the implementation of Nazi eugenic policies aimed at improving the “race” by eliminating individuals deemed as inferior. Many children diagnosed with psychiatric conditions or mental disabilities were murdered, including some who had been in Asperger's clinic. Asperger defended the social value of autistic psychopaths who had high intellectual abilities, but he did not do the same for those children with autistic traits and limited cognitive capacities (Czech, 2018; Sheffer, 2018). In the United States, children diagnosed with cognitive disabilities or various mental conditions, including autism, were often warehoused in inhumane institutions. Even when raised at home, they were stigmatized, had no access to education, and rarely could get adequate supports for their development. Thus, we cannot forget that psychological and psychiatric categories have often been used to establish hierarchies of social value that led to stigmatization, discrimination, and even murderous practices.

In the decades following Asperger's and Kanner's publications, psychiatrists debated the scientific usefulness of their new categories. Many clinicians argued that Kanner's infantile autism was a form of childhood schizophrenia. In some writings, and under pressure from his colleagues, Kanner himself accepted that autism could be classified as an early type of psychopathology within the family of the schizophrenias (Vicedo, 2021).

In addition, the relationship between Kanner's and Asperger's syndromes was unclear. Kanner and Asperger maintained that they had identified two different conditions. American psychologist Bernard Rimland, himself the father of an autistic son, emphasized the uniqueness of Kanner's syndrome to support the view that it was not related to childhood schizophrenia (Rimland, 1964). In Europe, the Dutch psychiatrist Dirk A. van Krevelen argued that Asperger had identified a personality type whereas Kanner was dealing with children who suffered from some form of brain damage that caused cognitive as well as affective impairments. He proposed using the term autism only for Kanner's syndrome (van Krevelen, 1962, 1971). However, he also posited that the two conditions might not be completely unrelated, as he encountered several families with one child diagnosed with Kanner's syndrome and another with Asperger's (van Krevelen, 1963).

While scientific experts were often trying to establish the boundaries between the two conditions, mothers of the first generation of children diagnosed with these conditions pointed out that their children did not fit neatly into one category or the other. In the United States, parents of autistic children founded the National Association for Autistic Children in 1965. Getting together at the association's annual meetings, and often accompanied by their children, these parents shared experiences and learned about each other's children. Many mothers, such as Clara Park, fought to have their knowledge about their children's development recognized (Park, 1967). Indeed, many parents became autism experts (Eyal et al., 2010; Silverman, 2012; Vicedo, 2021). Ruth Sullivan, the association's first elected president questioned the sharp differentiation between Kanner's and Asperger's syndromes. Sullivan, Park, and other mothers noticed that there was great variability among their children and that the categories put forth by Kanner and Asperger overlapped more than was previously believed. Kanner's syndrome often referred to children with very limited language abilities, whereas Asperger's syndrome was used for children who had some social disabilities but often had remarkable skills in other areas. Yet when the children grew up, those boundaries became blurred. Some children initially diagnosed with Kanner's syndrome were later indistinguishable from others diagnosed with Asperger's syndrome. Thus, the mothers discovered that autistic children developed in different ways and that autistic traits were not static over their life span (Vicedo, 2021).

Increasingly, scientific research also showed considerable diversity among autistic people. Kanner's follow up studies of the children described in his first papers revealed an enormous variability of outcomes (Kanner, 1971). In England, Victor Lotter's epidemiological studies uncovered “great differences” among children diagnosed as autistic (Lotter, 1966; see Evans, 2017 for changes in the conceptualization of autism in England). In 1981, Lorna Wing, a British psychiatrist and mother of an autistic daughter, proposed the notion of autism as a continuum (now referred to as a spectrum) (Wing, 1981).

In its diagnostic manual, the American Psychiatric Association followed, always belatedly, changing conceptions of autism. In 1952, the first edition of the Diagnostic and Statistical Manual of Mental Disorders (DSM-I) used the term “autism” to refer to a “childhood schizophrenic reaction” (American Psychiatric Association, 1952). “Infantile Autism” was introduced as a diagnosis separate from schizophrenia only in the DSM-III, published in 1980 (American Psychiatric Association, 1980). A few years later, in its 1987 revised edition, the DSM-IIIR changed the title of the diagnosis to “Autistic Disorder” (American Psychiatric Association, 1987). In 1994, the DSM-IV included four subcategories under Autistic Disorder: Asperger's Disorder, Pervasive Developmental Disorder not otherwise specified, Rett's Disorder, and Childhood Disintegrative Disorder (American Psychiatric Association, 1994). Then, the 2013 DSM-V eliminated them and established a unique category: Autism Spectrum Disorder (American Psychiatric Association, 2013; see Volkmar and Reichow, 2013, for specific changes in the different editions).

Today, the notion of autism as a spectrum is widely accepted, but its value as a diagnostic category is also widely contested.

## Discussion

Autism spectrum (AS) is an umbrella category that covers people with different abilities and impairments, and this poses several challenges. Many studies highlight the heterogeneity of autism and note the difficulties such diversity raises for scientific research, the design of suitable interventions, and the very conceptualization of autism (Szartmari, 1999; Thomas and Boellstorff, 2017; Happé and Frith, 2020; Hong et al., 2020; Mottron and Bzdok, 2020; Hughes, 2021; Nordahl et al., 2022; Rabot et al., 2023; Rentergem et al., 2023). Some scholars talk about “multiple” autisms (Happé et al., 2006; Singh, 2015). Many autistic people also find that those on one end of the spectrum are very different from those on the other end (Giles, 2014; Linton et al., 2014; Dyck and Russell, 2020). In addition, AS has boosted the visibility of those on the ‘higher' end of functionality (Russell et al., 2019; Mottron and Bzdok, 2020). Indeed, in most popular representations of autism, autistic people with serious impairments or cognitive disabilities are practically absent. Movies and television focusing on brilliant autistic people have rendered them invisible.

Within this context, the Lancet Commission's recommendation to use “profound autism” for administrative purposes represents an attempt to emphasize that different autistic people have distinctive psychological and medical needs, while still maintaining a unified notion of autism. The Commission's proposal aims to retain only one diagnostic category while establishing different subtypes at the level of care. It offers a pragmatic solution to the long-standing problem of how to address the heterogeneity of autism. Could it work?

In my view, the Commission's proposal is valuable at two main levels. One, it emphasizes the need to pay more attention to autistic people with deep cognitive disabilities. Since the time of Kanner and Asperger, these individuals have typically received less attention and support. Two, it concentrates on care. Employing categories for care seems a promising way to differentiate the distinct needs of individuals on the AS.

Autism is a capacious and evolving concept. First introduced to label a symptom of a mental illness, it was then adopted to name a syndrome or cluster of symptoms that could identify both a personality type and a psychopathology. Lately, many autistic people have claimed it as an identity. Through all those changes, the heterogeneity of autism has posed scientific and social challenges.

Today, this same heterogeneity also offers us an extraordinary opportunity to develop better scientific, clinical, and social practices. Though heterogeneity makes it difficult to establish neat categories, recognizing the great diversity among autistic people has led to more inclusive scientific and educational practices and a greater understanding and acceptance of autism. Different stakeholders have played key roles in bringing about those changes: scientists, parents, caretakers, and autistic people. After years of blaming mothers, many psychologists and psychiatrists ended up recognizing their valuable contributions for understanding autism (Vicedo, 2021). In recent years, thanks to autistic activists, the scientific community has acknowledged the importance of centering autistic people's voices and valuing their lived experience (Grinker, 2007; Bascom, 2012; Davidson and Orsini, 2013; Gillespie-Lynch et al., 2017; Fletcher-Watson et al., 2019). To make further progress continued dialogue among those stakeholders is imperative. Embracing the heterogeneity of autism as an opportunity means appreciating that autistic people have diverse needs and can make different types of contributions.

Categories, as history shows, shape lived experience. But attending carefully to lived experience - historical and contemporary - can help us to develop better categories, ones that foster de-stigmatization and do justice to the diverse social, psychological, and medical needs of all autistic people. It is an opportunity we should not squander.

## Author contributions

The author confirms being the sole contributor of this work and has approved it for publication.
